# First-principles pressure-dependent investigation of the physical and superconducting properties of ThCr_2_Si_2_-type superconductors SrPd_2_X_2_ (X = P, As)

**DOI:** 10.1098/rsos.241435

**Published:** 2024-12-18

**Authors:** Md. Asrafusjaman, M. A. Islam, Areef Billah, Bashir Ahmmad

**Affiliations:** ^1^ General Education Department, City University, Birulia, Savar, Dhaka 1340, Bangladesh; ^2^ Department of Physics, University of Barishal, Barisal, Bangladesh; ^3^ Graduate School of Science and Engineering, Yamagata University, Yamagata, Japan

**Keywords:** type II superconductor, density functional theory, crystal structure, Debye temperature, superconducting properties

## Abstract

The physical and superconducting characteristics of SrPd_2_P_2_ and SrPd_2_As_2_ compounds with applied pressure were calculated using density functional theory. The pressure effect on the structural properties of these compounds was investigated. The results show that both lattice constants and volume decrease almost linearly with increasing pressure. The elastic constants (*C*_ij_) for both compounds increase with increasing pressure and satisfy Born criteria for mechanical stability. The elastic parameters indicate the ductile behaviour and anisotropic nature of these compounds under applied pressure. The Debye temperature (*ϴ*_D_) and melting temperature (*T*_m_) increase with increasing pressure. The electronic band structure calculation of both compounds exhibits metallic characteristics at different pressures. The density of electronic states at the Fermi level, *N*(*E*_F_), consistently decreases as pressure increases, which is also reflected in the repulsive Coulomb pseudopotential (µ*), and the electron–phonon coupling constant (*λ*). These optical features suggest that both compounds are suitable for optoelectronic device applications. Furthermore, the superconducting transition temperature, *T*_c_, for both compounds is predicted to vary with applied pressure due to changes in *ϴ*_D_ *, N*(*E*_F_), µ* and *λ*.

## 1. Introduction

Since its discovery in mercury in 1911, superconductivity, a remarkable quantum phenomenon, has fascinated scientists due to its defining properties of perfect diamagnetism and zero electrical resistance. The quest to discover new superconductors with higher critical temperatures (*T*_c_) for practical applications has driven significant research in this field [1]. Superconducting materials are generally classified into three main groups based on their parent compounds: conventional (metallic elements or alloys), possibly unconventional (magnesium diboride, bismuthate superconductors, etc.) and unconventional (cuprate superconductors, heavy fermion superconductors and iron-based superconductors) [2]. Among these, iron-based 122-type pnictides have become a central focus of superconductivity research due to their unique properties, including high transition temperatures, *T*_c_ and lower electromagnetic anisotropy compared with other high-*T*_c_ superconductors such as cuprates [3]. These characteristics make them highly suitable for use in electronic devices. Furthermore, their relatively simpler crystal structure compared with cuprate superconductors allows for easier fabrication and investigation, facilitating experimental studies [4,5]. This combination of high performance and ease of synthesis positions iron-based 122-type pnictides as promising candidates for advancing superconducting technology. However, iron-based 122-type pnictides face challenges related to their susceptibility to doping and the presence of magnetic impurities, which can impair their superconducting properties [6,7]. To overcome these obstacles, scientists have been exploring pnictides without iron, which exhibit unique electrical characteristics that could potentially address these limitations. The absence of iron may reduce magnetic impurities, enhancing the stability and durability of the superconducting state. Additionally, the distinct crystal structures of iron-free pnictides offer more favourable pathways for electron pairing, which is crucial for achieving superconductivity at higher temperatures. Chemical substitutions that can fine-tune their electronic properties provide another promising approach to optimizing their superconducting characteristics [5,8,9]. One significant class of materials under study is the family of ternary intermetallic compounds, which is defined by the chemical formula: AM_2_X_2_, where A = lanthanide or alkaline earth elements; M = transition metals and X = P and As, exhibiting the ThCr_2_Si_2_-type body-centred tetragonal structures with space group *I*4*/*mmm. This family is the biggest known, consisting of almost 700 members of the 122-type intermetallic compounds (so-called 122 phases) [10]. Recently, the ThCr_2_Si_2_-type structures have received significant attention from researchers due to their fascinating properties, including superconductivity at both low [11,12] and high *T*_c_ [13], pressure-induced superconductivity [14–17], doping-induced superconductivity [18] and various magnetic properties [19,20]. This study focuses on two low *T*_c_ members of the 122 family of the ThCr_2_Si_2_-type structures namely, SrPd_2_As_2_ and SrPd_2_P_2_ superconductors [21,22]. The crystallographic, electronic transport, thermal, magnetic and superconducting properties of SrPd_2_As_2_ and SrPd_2_P_2_ have been explored through experimental investigations. In these investigations, the superconducting transition temperatures were determined from zero-field specific heat *C*_p_(T) measurements, with *T*_c_ 0.92 K for SrPd_2_As_2_ and *T*_c_ 0.7 K for SrPd_2_P_2_. These superconductors also provide an ideal playground for studying the role of crystal structure for quenching magnetism and generating ambient pressure superconductivity [21,22]. Karaca *et al*. theoretically investigated and observed that the *T*_c_ is 2.05 K for SrPd_2_As_2_ [23]. Low-temperature superconductivity in SrPd_2_P_2_ was theoretically demonstrated in 2021 by Islam and Hossain and they observed that the *T*_c_ is 0.10 K [24]. A theoretical investigation is required to explore the physical characteristics and superconducting behaviour of SrPd_2_P_2_ and SrPd_2_P_2_ under pressure. To the best of our extensive survey, the fundamental physical properties and superconducting nature of both SrPd_2_P_2_ and SrPd_2_As_2_ superconductors have not yet been theoretically explored in the context of applied pressure. Theoretical Debye temperatures (*ϴ*_D_) from elastic moduli of SrPd_2_P_2_ and SrPd_2_As_2_ have not been reported under pressure. One of the most efficient and clean thermodynamic ways of changing the properties of a compound is to apply pressure. The applied pressure may increase the value of *T*_c_ to high-temperature superconductivity, especially for low-temperature superconductors. In recent years, a large number of research projects have been performed under pressure to achieve high-temperature superconductivity near room temperature [25–28]. ThCr₂Si₂-type (122-type) superconductors also exhibit a decrease in *T*_c_ with applied pressure [16,17]. Islam *et al*. exhibited the pressure-dependent nature of *ϴ*_D_ in low-temperature superconductor CaPd_2_P_2_ and predicted the possible variation of *T*_c_ with applied hydrostatic pressure using density functional theory (DFT)-based *ab initio* calculations [29]. Asrafusjaman *et al*. also investigated the pressure-induced effect on the superconducting properties of non-centrosymmetric superconductor TaRh_2_B_2_ and NbRh_2_B_2_ compounds using DFT calculation. They predicted the *T*_c_ of both compounds to vary with the applied pressure due to an increase in *ϴ*_D_ [30]. In 2020, Mridha & Naqib theoretically investigated the BaM_2_P_2_ (M = Ni, Rh) superconductor. They predicted the pressure-dependent characteristics of *T*_c_ decrease due to the increase in *ϴ*_D_ and the decrease in *N*(*E*_F_) [31]. Recently, Alam *et al*. exhibited the pressure effect on KB_2_H_8_. They predicted the pressure-dependent characteristics of *T*_c_ to decrease with a pressure-induced variation in the *ϴ*_D_ and decreasing *N*(*E*_F_) [32]. These research articles motivated us to carry out DFT-based *ab initio* calculations on the physical and superconducting characteristics of SrPd_2_P_2_ and SrPd_2_As_2_ under pressure. The mechanical parameters such as ductility/brittleness and hardness/softness are required to determine the suitability of a new material for the fabrication of devices. It is also required to justify the Born elastic stability conditions of a compound to be mechanically stable. Therefore, it is essential to disclose the Debye temperature value, especially for superconductors. The melting temperature is a crucial thermodynamic characteristic indicating a material’s suitability for high-temperature applications. The optical functions, such as reflectivity, optical absorption, conductivity and dielectric functions, are crucial parameters for predicting the application of a compound from various perspectives. The literature review reveals a lack of theoretical investigation into the fundamental physical and superconducting properties of ThCr_2_Si_2_-type SrPd_2_P_2_ and SrPd_2_As_2_ compounds under pressure. Therefore, this study focuses on a comprehensive theoretical investigation of the structural, mechanical, thermodynamic, electronic, optical and possible variations of *T*_c_ in SrPd_2_P_2_ and SrPd_2_As_2_ superconductors under pressure up to 16 GPa using DFT.

## 2. Computational details

In this study, the CASTEP (Cambridge Serial Total Energy Package) simulation code [33] was used to calculate the ground state properties of SrPd_2_P_2_ and SrPd_2_As_2_ compounds [34,35]. The exchange–correlation interaction was described by the generalized gradient approximation (GGA) following Perdew–Burke–Ernzerhof (PBE) [36]. Vanderbilt-type ultra-soft pseudopotential [37] was used to describe the interaction between ions and electrons for all atoms. Pseudo-atomic calculations were performed for the valence electrons of Sr-4s^2^ 4p^6^ 5s^2^, Pd-4d^10^ and P-3s^2^ 3p^3^ in the case of SrPd_2_P_2_ superconductor and Sr-4s^2^ 4p^6^ and 5s^2^, Pd-4d^10^ and As-4s^2^ 4p^3^ in the case of SrPd_2_As_2_ superconductor. The Broyden–Fletcher–Goldferb–Shanno (BFGS) algorithm was used to perform the geometry optimization of SrPd_2_P_2_ and SrPd_2_As_2_ compounds [38]. The conversing cut-off energy of a plane wave of 750 eV is performed for all calculations, including geometry optimization, density of states (DOS), band structure, charge density difference and optical properties for both compounds. For the k-point sampling of the Brillouin zone, 16 × 16 × 8 was used with the help of the Monkhorst–Pack scheme [39]. Geometry optimization for SrPd_2_P_2_ and SrPd_2_As_2_ compounds was performed with a convergence tolerance energy of 1 × 10^−5^ eV/atom, a maximum force of 0.03 eV Å^−1^, a maximum stress of 0.05 GPa and a maximum displacement of 0.001 Å. On the other hand, the stress–strain method [40] was employed for the determination of the elastic stiffness constants of both compounds. To calculate the elastic properties, the strain amplitude was selected at 0.003. Furthermore, the convergence tolerance energy of 4.0 × 10^-6^ eV/atom, the maximum force of 0.01 eV Å^−1^ and the maximum displacement of 4.0 × 10^−4^ Å were adjusted.

## 3. Results and discussion

### 3.1. Structural properties

**Figure 1.**
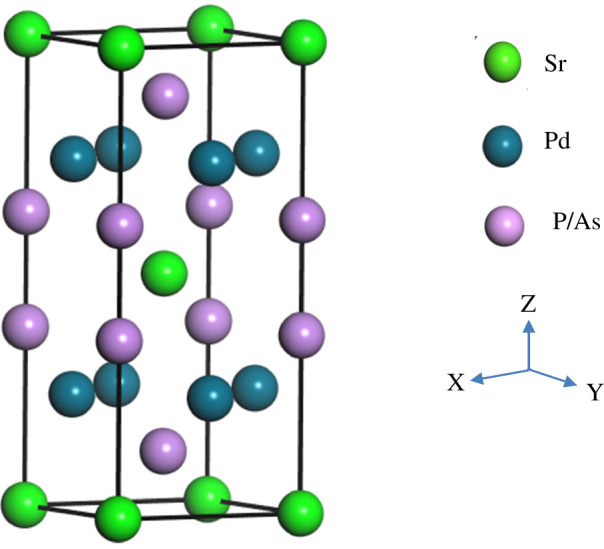
Schematic crystal structure of SrPd_2_X_2_ (X = P/As) compound.

The single-crystalline superconducting SrPd_2_X_2_ (X = P and As) compounds have a body-centred structure and belong to the renowned ThCr_2_Si_2_-type family with space group *I*4*/*mmm (no. 139) [21,22]. The crystal structure of SrPd_2_X_2_ is drawn by implementing VESTA software, presented in figure 1 [41]. The Wyckoff sites occupied by the Sr and Pd atoms are 2a (0, 0, 0) and 4d (0, 0.5, 0.25), respectively, for both superconductors. On the other hand, P and As atoms possess Wyckoff sites 4e (0, 0, 0.386) and 4e (0, 0, 0.377), respectively. The optimized lattice parameters and cell volumes under different uniform pressures with the available theoretical and experimental data of both superconductors are tabulated in table 1. At 0 GPa, the optimized lattice parameters and cell volumes of both compounds using DFT are in good agreement with the experimental and theoretical data [21–24]. The structural properties of SrPd_2_P_2_ and SrPd_2_As_2_ compounds are significantly influenced by the applied pressure.

**Table 1.** The computed values of lattice parameters and unit cell volume of SrPd_2_P_2_ and SrPd_2_As_2_ superconductor under pressure.

*P* (GPa)	phase	*a* (Å)	*c* (Å)	*V* (Å^3^)	reference
(exp.)	SrPd_2_P_2_	4.241	9.710	174.645	[21]
	SrPd_2_As_2_	4.376	10.167	194.691	[20]
0 (GGA)	SrPd_2_P_2_	4.153	10.232	176.475	[23]
	SrPd_2_As_2_	4.426	10.362	202.986	[22]
0	SrPd_2_P_2_	4.245	9.740	175.525	this study
	SrPd_2_As_2_	4.407	10.277	199.609	
4	SrPd_2_P_2_	4.193	9.654	169.703	this study
	SrPd_2_As_2_	4.343	10.153	191.458	
8	SrPd_2_P_2_	4.146	9.588	164.843	this study
	SrPd_2_As_2_	4.295	10.086	186.089	
12	SrPd_2_P_2_	4.106	9.530	160.659	this study
	SrPd_2_As_2_	4.226	10.003	178.663	
16	SrPd_2_P_2_	4.070	9.478	157.028	this study
	SrPd_2_As_2_	4.186	9.938	174.109	

**Figure 2.**
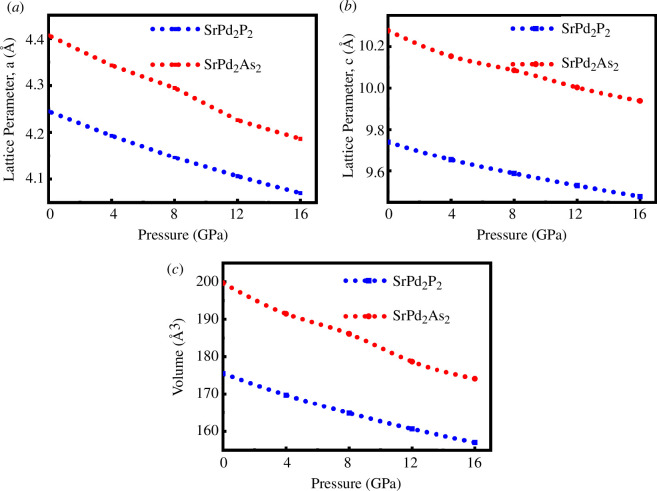
Pressure dependence of optimized (*a, b*) lattice parameters and (*c*) unit cell volume of SrPd_2_P_2_ and SrPd_2_As_2_ compounds at various pressures.

**Figure 3.**
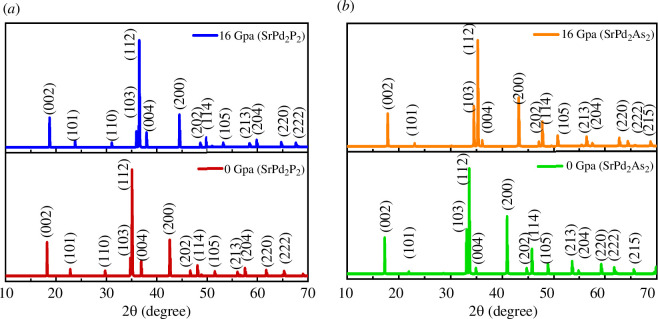
Simulated XRD pattern of the (*a*) SrPd_2_P_2_ and (*b*) SrPd_2_As_2_ compounds at various pressures.

Figure 2 depicts the effect of hydrostatic pressure on the lattice constants and unit cell volumes of both compounds. Figure 2 illustrates that the lattice parameters and the unit cell volumes decrease with increasing applied pressure, which implies that the space among the atoms is reduced with hydrostatic pressure. The reduction of the space among the atoms under pressure creates a repulsive effect, which helps to enhance the stiffness of the compound compression under applied pressure. Figure 3*a,b* illustrates the simulated X-ray diffraction (XRD) patterns for two different compounds, SrPd_2_P_2_ and SrPd_2_As_2_, under different pressures (0 GPa and 16 GPa), where the XRD data were generated by simulating an X-ray source [42] with a wavelength of 1.5406 Å from a copper (Cu) source. For both compounds, the diffraction patterns show clear peaks corresponding to specific crystallographic planes, such as (002), (101) and (112), both at 0 and 16 GPa. When the pressure increases to 16 GPa, the diffraction patterns shift slightly towards higher 2θ angles, suggesting compression of the crystal lattice. This shift reflects a decrease in the lattice constant as a result of the applied pressure. According to Bragg’s Law, the diffraction angle increases as the interplanar spacing decreases. Therefore, the slight shift of the diffraction lines from the original position corresponds to a decrease in the unit cell size, which is shown in figure 2.

### 3.2. Mechanical and elastic properties

Elastic constants provide detailed insights into the mechanical properties and bonding behaviour of solids. Elastic constants also provide crucial information about the nature of chemical bonding in solids, specifically offering insights into mechanical stability, material stiffness and response to external stresses of a material [43,44]. The tetragonal structure of SrPd_2_As_2_ and SrPd_2_P_2_ compounds has six different elastic constants: *C*_11_, *C*_12_, *C*_13_, *C*_33_, *C*_44_ and *C*_66_ [45]. Among the six independent elastic constants, *C*_11_ and *C*_33_ assess the material’s response to uniaxial stresses, with *C*_11_ representing stiffness along the *x*-axis and *C*_33_ corresponding to the *z*-axis. On the other hand, *C*_44_ and *C*_66_ describe the response to shearing stresses. Specifically, *C*_44_ deals with shear deformation in planes perpendicular to the *z*-axis, while *C*_66_ addresses shear deformation within the *x–y* plane. *C*_12_ and *C*_13_ measure the material’s response to axial stress along one axis and strain along a perpendicular axis, where *C*_12_ describes how stress along the *x*-axis influences strain in the *y*-axis, and *C*_13_ handles the interaction between the *z*-axis and the *x*- or *y*-axes [31], which is tabulated in table 2.

**Table 2.** The computed value elastic constants (*C*_11_, *C*_12_, *C*_13_, *C*_33_, *C*_44_ and *C*_66_) of SrPd_2_P_2_ and SrPd_2_As_2_ superconductor.

*P* (GPa)	phase	*C*_11_	*C*_12_	*C*_13_	*C*_33_	*C*_44_	*C*_66_
0	SrPd_2_P_2_	167.70	56.47	81.40	198.03	66.08	40.41
	SrPd_2_As_2_	158.74	42.03	70.47	132.37	45.62	24.36
4	SrPd_2_P_2_	200.46	76.95	100.46	227.43	74.23	47.41
	SrPd_2_As_2_	180.19	48.03	84.14	154.69	56.03	27.99
8	SrPd_2_P_2_	223.58	89.22	116.03	247.48	82.20	55.04
	SrPd_2_As_2_	180.87	75.31	94.67	198.46	53.18	32.44
12	SrPd_2_P_2_	247.44	103.029	132.074	274.04	89.85	62.84
	SrPd_2_As_2_	230.65	82.06	118.28	233.05	64.36	40.81
16	SrPd_2_P_2_	269.76	115.94	146.93	300.91	96.88	70.32
	SrPd_2_As_2_	255.91	103.54	137.92	258.45	67.90	46.47

From table 2, it is evident that at all pressures, *C*_33_ > *C*_11_ for the SrPd_2_P_2_ compound. This indicates that the material exhibits greater resistance to deformation along the z-axis compared to the x-axis, highlighting its stiffer nature along the z-axis. In contrast, for SrPd₂As₂, *C*_11_ > *C*_33_ at 0–4 GPa, suggesting that the material is stiffer in the x-y plane than along the z-axis in terms of uniaxial stiffness. However, as the pressure increases to 8–16 GPa, *C*_33_ *> C*_11_ *,* indicating a transition to greater stiffness along the z-axis and highlighting enhanced resistance to deformation in this direction under higher pressures.

Table 2 also shows that *C*_44_ > *C*_66_ under applied pressure, which indicates that both materials exhibit greater resistance to shear deformation along planes perpendicular to the *z*-axis compared with shear within the *x–y* plane under applied pressure. All the qualitative and quantitative characteristics of *C_ij_* clearly reflect the layered nature of the crystal structure under study. The values of *C_ij_* make it evident that the chemical bonds within the *ab*-plane are stronger than those along the *c*-direction under applied pressure. The Born’s mechanical stability criterion for the tetragonal structure without pressure condition can be expressed as [45]


(3.1) $$\begin{array}{l} C_{11}>0,\;C_{33}>0,\;C_{44}>0,C_{66}>0,\\ C_{11}-C_{12}>0,\;C_{11}+C_{33}-2C_{13}>0,\\ 2\left(C_{11}+C_{12}\right)+C_{33}+4C_{13}>0. \end{array}$$

The calculated elastic constants of the tetragonal crystal are listed in table 2. The listed elastic constants are positive, and the mechanical stability conditions of tetragonal crystals indicate that SrPd_2_As_2_ and SrPd_2_P_2_ compounds are mechanically stable. For the tetragonal crystal, Born’s mechanical stability under pressure conditions can be observed from the following equations [46–48]:


(3.2) $$\begin{array}{l} \;C_{44}-P>0,C_{66}-P>0,\\ C_{11}-C_{12}-2P>0,\;\\ \left(C_{33}-P\right)\left(C_{11}+C_{12}\right)-2{\left(C_{13}+P\right)}^{2}>0. \end{array}$$

Table 2 shows that the current calculated results satisfy the above conditions, indicating the mechanical stability of the SrPd_2_As_2_ and SrPd_2_P_2_ superconductors under applied pressure. The most significant mechanical parameters of solids, as well as the bulk modulus (*B*), shear modulus (*G*), Young’s modulus (*E*), Poisson’s ratio (ν), universal anisotropy (*A* ^U^) and Vickers hardness (*H*_V_), can be evaluated by using the Voigt–Reuss–Hill average schemes as follows [49–51]:


(3.3) $$B_{V}=\frac{2}{9}\left(C_{11}+C_{12}+2C_{13}+\frac{C_{33}}{2}\right),$$

(3.4) $$B_{R}=\frac{\left(C_{11}+C_{12})C_{33}-2C_{13}^{2}\right)}{C_{11}+C_{12}-4C_{13}+2C_{33}},$$

(3.5) $$G_{V}=\frac{\left(7C_{11}-5C_{12}-4C_{13}+2C_{33}+12C_{44}\right)}{30},$$

(3.6) $$G_{R}=\frac{15}{2}{\left[\frac{2C_{11}+2C_{12}+4C_{13}+C_{33}}{C_{33}\left(C_{11}+C_{12}\right)-2C_{13}^{2}}+\frac{3C_{11}-3C_{12}+6C_{44}}{C_{44}\left(C_{11}-C_{12}\right)-2C_{14}^{2}}\right]}^{-1},$$

(3.7) $$B=\frac{B_{V}+B_{R}}{2}$$

and


(3.8) $$G=\frac{G_{V}+G_{R}}{2}.$$

The following expressions are used to calculate the Young’s modulus (*E*) and Poisson’s ratio ( v ):


(3.9) $$E=\frac{9BG}{\left(3B+G\right)},$$

(3.10) $$v=\frac{3B-2G}{6B+2G},$$

where, *B*_V_ and *B*_R_ represent the Voigt and Reuss of bulk moduli, respectively. The Voigt and Reuss of shear moduli are represented by *G*_V_ and *G*_R_, respectively. The values that were found for the mean bulk modulus (*B*), mean shear modulus (*G*), Young’s modulus (*E*), Poisson’s ratio (*ν*), universal anisotropy (*A* ^U^) and Vickers hardness (*H*_V_) of compounds under applied pressures are tabulated in table 3.

**Table 3.** Pressure-induced variation of Voigt bulk modulus *B*_V_, Reuss bulk modulus *B*_R_, mean bulk modulus *B*, Voigt shear modulus *G*_V_, Reuss shear modulus *G*_R_, mean shear modulus *G* , Young’s modulus *E* (GPa) of SrPd_2_P_2_ and SrPd_2_As_2_ superconductor.

*P* (GPa)	phase	*B*_V_	*B*_R_	*B*	*G*_V_	*G*_R_	*G*	*E*
0	SrPd_2_P_2_	107.99	105.69	106.84	55.46	53.30	54.38	139.48
	SrPd_2_As_2_	90.64	90.64	90.64	40.91	37.12	39.01	102.36
4	SrPd_2_P_2_	131.57	129.85	130.71	62.54	60.51	61.52	159.54
	SrPd_2_As_2_	105.30	105.17	105.24	47.93	42.94	45.44	119.16
8	SrPd_2_P_2_	148.58	146.91	147.75	68.78	66.63	67.70	176.20
	SrPd_2_As_2_	103.36	101.28	102.32	50.43	47.18	48.80	126.32
12	SrPd_2_P_2_	167.03	165.17	166.10	75.29	73.09	74.19	193.73
	SrPd_2_As_2_	147.95	146.87	147.41	58.95	56.30	57.63	152.95
16	SrPd_2_P_2_	184.45	182.30	183.38	81.53	79.30	80.41	210.47
	SrPd_2_As_2_	169.89	168.96	169.43	62.51	60.47	61.49	164.57

Table 3 shows that the values of *B*, *G* and *E* for both compounds increase as the applied pressure increases, with the exception of the bulk modulus value for SrPd_2_As_2_ at 8 GPa. Such an increase is natural since applied pressure makes the crystals of both compounds stiffer. The higher (lower) value of *B*, *G* and *E* implies the hard (soft) nature of a material [52,53]. Table 3 reveals that the computed values of *B, G* and *E* of SrPd_2_P_2_ are higher than those of SrPd_2_As_2_ under applied pressure up to 16 GPa, which indicates that SrPd_2_P_2_ is harder than SrPd_2_As_2_ under applied pressure. The ratio between *B* and *G* moduli is known as Pugh’s ratio (*B/G*). A large value (greater than 1.75) of this ratio indicates ductile behaviour, whereas a low value (less than 1.75) indicates the brittleness of a material [30].

As shown in table 4, the evaluated Pugh’s ratio of both compounds reveals ductile behaviour under applied pressure that is higher than the critical values. SrPd_2_As_2_ exhibits a higher Pugh ratio value compared with SrPd_2_P_2_, which indicates that the SrPd_2_As_2_ compound has a high ductility nature under the investigated pressures. At 16 GPa, the maximum value of the Pugh ratio is observed at 2.76 for SrPd_2_As_2_ and 2.28 for SrPd_2_As_2_. These results suggest that applying hydrostatic pressure can enhance the ductility behaviour of these compounds. The bonding forces of solids appear from the values of Poisson’s ratio, and the central force of solids is considered between 0.25 and 0.50 [54]. In this study, the calculated value of both compounds’ Poisson’s ratio was within the range of 0.25 and 0.50 under pressures, indicating the presence of strong central forces among the atoms of these compounds. The universal anisotropy factor (*A* ^U^) is a significant parameter to reveal the anisotropic nature. It can be evaluated using the following equation [55]:

**Table 4.** The calculated *B/G* ratio, Poisson’s ratio (*v*), elastic anisotropic factor (*A* ^U^), Vickers hardness (*H*_v_) average sound velocity *V*_M_ (m s^−1^), Debye temperature *ϴ*_D_ (K) and melting temperature (*T*_m_) of SrPd_2_P_2_ and SrPd_2_As_2_ superconductor.

*P* (GPa)	phase	*B/G*	*v*	*A* ^U^	*H*_V_	*v*_m_ (m s^−1^)	θ_D_(K)	*T*_m_ (K)
0	SrPd_2_P_2_	1.95	0.28	0.22	6.40	3129.76	358	1154
	SrPd_2_As_2_	2.32	0.31	0.51	3.36	2534.97	278	1029
4	SrPd_2_P_2_	2.21	0.30	0.18	6.22	3281.817	380	1295
	SrPd_2_As_2_	2.31	0.31	0.58	3.98	2679.78	298	1127
8	SrPd_2_P_2_	2.18	0.30	0.17	6.45	3395.93	397	1396
	SrPd_2_As_2_	2.10	0.29	0.36	5.18	2737.96	307	1159
12	SrPd_2_P_2_	2.24	0.30	0.16	6.68	3512.35	414	1507
	SrPd_2_As_2_	2.56	0.33	0.24	4.14	2934.66	334	1396
16	SrPd_2_P_2_	2.28	0.30	0.15	6.92	3617.30	430	1615
	SrPd_2_As_2_	2.76	0.34	0.17	3.80	2999.98	344	1509


(3.11) $$A^{U}=\frac{5G_{V}}{G_{R}}+\frac{B_{V}}{B_{R}}-6.$$

**Figure 4.**
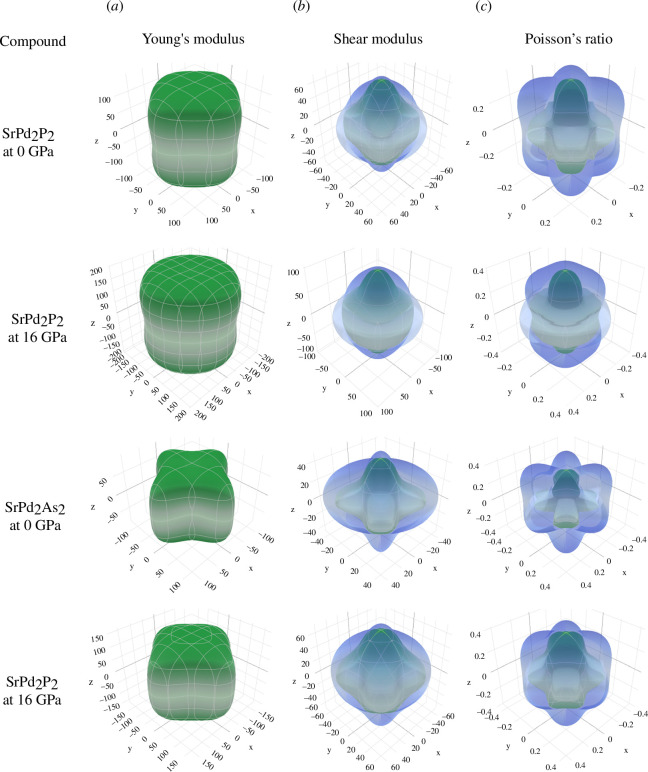
Three-dimensional representation of (*a*) Young’s modulus, (*b*) shear modulus and (*c*) Poisson’s ratio of SrPd_2_P_2_ and SrPd_2_As_2_ at 0 GPa and 16 GPa.


The value of *A* ^U^ is equal to zero for isotropic crystals, and any fluctuations from zero indicate the anisotropic nature of a material. From table 4, the values of the *A* ^U^ indicate that the SrPd_2_P_2_ and SrPd_2_As_2_ compounds are anisotropic in nature under applied pressure. Three-dimensional visualization of the directional dependencies of Young’s modulus (*E*), shear modulus (*G*) and Poisson’s ratio (*υ*) can provide further insights regarding the isotropic/anisotropic nature. The elastic components’ spherical and circular nature indicates that the materials are isotropic, whereas the deviation from the spherical/circular nature implies an anisotropic nature. The *E*, *G* and *υ* of SrPd_2_P_2_ and SrPd_2_As_2_ compounds at 0 and 16 GPa are presented three-dimensionally in figure 4, where ELATE code has been used to plot the three-dimensional images [56]. The three-dimensional plots clearly reveal that *E*, *G* and *υ* of both compounds are found to be anisotropic at 0 and 16 GPa. The Vickers hardness (*H*_V_) of a material can be calculated by a novel theoretical model developed by Chen *et al.* as follows [57]:

(3.12) $$H_{V}=2{\left(K^{2}G\right)}^{0.585}-3.$$

Table 4 shows the calculated values of Vickers hardness (*H*_v_) for these compounds. The values of *H*_v_ of the SrPd_2_P_2_ compound increase with increasing applied pressure without exception at 4 GPa. The highest value of *H*_v_ is found at 16 GPa for the SrPd_2_P_2_ compound. On the other hand, the value of *H*_v_ of the SrPd_2_As_2_ compound gradually changes under applied pressure. SrPd_2_As_2_ exhibits a lower value of *H*_v_ compared with SrPd_2_P_2_ under applied pressure, which implies that SrPd_2_P_2_ has much higher hardness than SrPd_2_As_2_ under applied pressure.

### 3.3. Thermodynamic properties

To improve the performance of superconductors in real-world applications, it is essential to comprehend features like thermodynamics and make predictions about how they will behave in various conditions. The investigation of thermodynamic properties is essential to determine the numerous solid-state phenomena of crystals. This section provides an in-depth analysis of key thermodynamic properties, including the Debye temperature (*ϴ*_D_) and melting temperature (*T*_m_), of SrPd_2_P_2_ and SrPd_2_As_2_ compounds under pressures up to 16 GPa. The Debye temperature (*ϴ*_D_) is a vital thermodynamic parameter that affects properties such as specific heat, lattice vibrations, thermal expansion and *T*_m_. In superconducting materials, *ϴ*_D_ is especially important due to its direct relationship with *T*_c_ [30], which will be explained later. The maximum frequency mode of vibrations depends on the *ϴ*_D_ value, and it can be estimated using various methods. Here, *ϴ*_D_ is calculated using isotropic sound velocities such as transverse sound velocity *v*_t_, longitudinal sound velocity *v*_l_ and average sound velocity *v_m_* using the following formulae [58]:


(3.13) $${ϴ}_{D}=\;\frac{h}{k_{B}}{\left[\frac{3n}{4\pi }\left(\frac{N_{A}\rho }{M}\right)\right]}^{\frac{1}{3}}v_{m},$$

(3.14) $$v_{m}=\;{\left[\frac{1}{3}\left(\frac{2}{v_{t}^{3}}+\frac{1}{v_{l}^{3}}\right)\right]}^{-\frac{1}{3}},$$

(3.15) $$v_{l}={\left(\frac{3B+4G}{3\rho }\right)}^{\frac{1}{2}}$$

and


(3.16) $$v_{t}={\left(\frac{G}{\rho }\right)}^{\frac{1}{2}},$$

where *k*_B_, *h*, *N*_A_, *M*, *n* and *ρ* denote the Boltzmann constant*,* Planck constant, Avogadro’s number, molecular weight, atom number in the molecule and density, respectively. The computed values of *ϴ*_D_ under various hydrostatic pressures are presented in table 4. From table 4, it can be concluded that the value of *ϴ*_D_ of SrPd_2_P_2_ and SrPd_2_As_2_ compounds increases with increasing pressure up to 16 GPa, and the maximum *ϴ*_D_ values are observed at 430 and 344 K for SrPd_2_P_2_ and SrPd_2_As_2_ at 16 GPa. This suggests that the materials become more resistant to thermal vibrations at higher pressures. SrPd_2_P_2_ has a higher *ϴ*_D_ than SrPd_2_As_2_ at each corresponding pressure, indicating stiffer lattice vibrations in SrPd_2_P_2_ compared with SrPd_2_As_2_. *T*_m_ is another crucial thermodynamic parameter that limits the application of a material at high temperature. *T*_m_ indicates the temperature at which a solid becomes a liquid under specific pressure conditions. *T*_m_ of the tetragonal crystal can be evaluated by the elastic constants *C*_ij_ data as follows [59]:


(3.17) $$T_{m}=354+4.5\;\frac{2C_{11}+\;C_{33}}{3}.$$

Table 4 presents *T*_m_ for two compounds, SrPd_2_P_2_ and SrPd_2_As_2_ superconductors, at various pressures. From table 4, the value of *T*_m_ of both compounds increases with applied pressure, which is a result of the increasing trend of *C*_11_ and *C*_33_ values with applied pressure (table 2). Both compounds exhibit a clear trend of increasing *T*_m_ with increasing pressure, indicating that higher pressures stabilize the solid phases. For SrPd_2_P_2_ and SrPd_2_As_2_ superconductors, the maximum *T*_m_ is 1615 and 1509 K at 16 GPa, respectively. At all measured pressures, SrPd_2_P_2_ consistently shows higher melting temperatures than SrPd_2_As_2_, suggesting stronger bonding and higher thermal stability in SrPd_2_P_2_.

### 3.4. Electronic band structure

**Figure 5.**
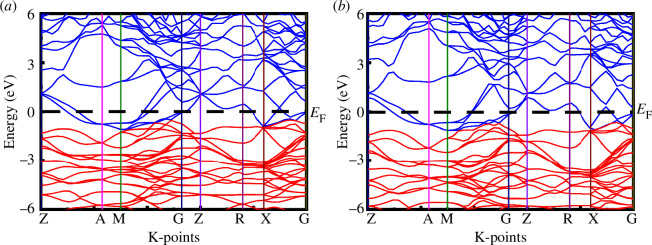
Electronic band structure of SrPd_2_P_2_ at (*a*) 0 GPa and (*b*) 16 GPa.

**Figure 6.**
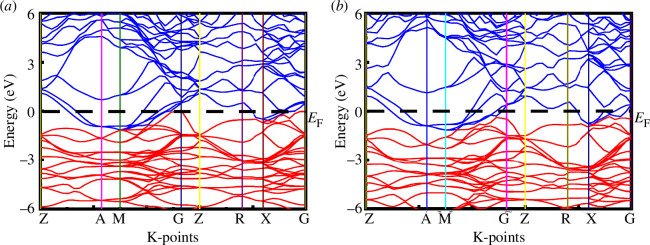
Electronic band structure of SrPd_2_As_2_ at (*a*) 0 GPa and (*b*) 16 GPa.

**Figure 7.**
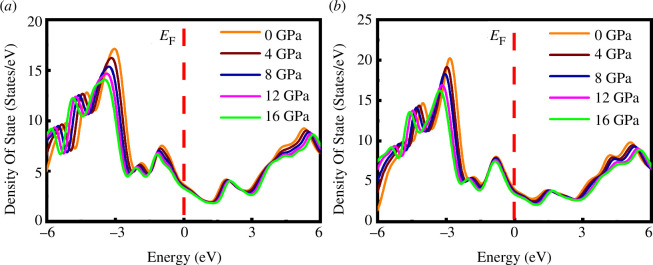
Total density of states of (*a*) SrPd_2_P_2_ and (*b*) SrPd_2_As_2_ at various pressures.

The electronic band structure is a crucial aspect that sheds light on the electronic, optical and magnetic properties of materials at the microscopic level. In this analysis, the electronic band structure of SrPd_2_P_2_ and SrPd_2_As2 at 0 GPa and 16 GPa pressure, along the high-symmetry directions in the Brillouin zone, is illustrated in figures 5 and 6. The horizontal dashed black line at the zero-energy point signifies the Fermi level (*E*_F_), which separates the valence band and conduction band. The red lines signify the valence bands, which correspond to the energy states occupied by electrons at lower energies. These bands are positioned below the Fermi level, showing they are fully occupied. On the other hand, the blue lines represent the conduction bands, which are higher energy states accessible to electrons when they gain sufficient energy to surpass the Fermi level. From figures 5 and 6, the conduction bands (blue) intersect or approach the Fermi level, especially between the high-symmetry points G and X. This intersection indicates the availability of electron states at the Fermi level, allowing electrons to transition easily between the valence and conduction bands. Such behaviour is characteristic of metallic properties, as the availability of electron states at *E*_F_ enhances electrical conductivity. The total density of states (TDOS) has been investigated in depth to gain a better understanding of the electrical characteristics and the metallic behaviour of SrPd_2_P_2_ and SrPd_2_As_2_ compounds. Figure 7 represents the calculated TDOS of SrPd_2_P_2_ and SrPd_2_As_2_ above and below the *E*_F_. The vertical dash line represents *E*_F_. The calculated value of TDOS at *E*_F_ is 1.83 states eV^−1^ f.u.^−1^ for SrPd_2_P_2_ and 2.02 states eV^−1^ f.u.^−1^ for SrPd_2_As_2_, which are in good agreement with the earlier study at 0 GPa [23,24].

The density of states at the Fermi level, *N*(*E*_F_) is a crucial electronic parameter that influences many charge transport and magnetic characteristics of metals. It can determine the repulsive Coulomb pseudopotential, μ*, which can be expressed by the following equation [60–62]:


(3.18) $$\mu^{*}=\;0.26\frac{N\left(E_{F}\right)}{1+N\left(E_{F}\right)}.$$

**Figure 8.**
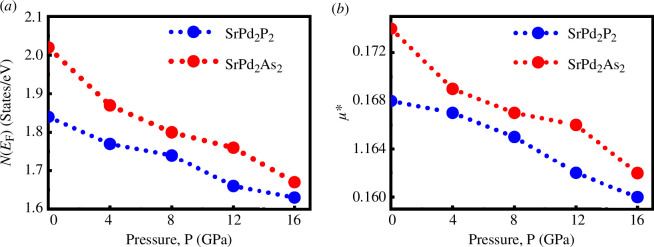
Pressure dependence of (*a*) electronic density of states at the Fermi level *N*(*E*_F_) (states eV^−1^) and (*b*) repulsive Coulomb pseudopotential µ* of SrPd_2_P_2_ and SrPd_2_As_2_ compounds.

The density of states at the Fermi level, *N(E_F_)* and repulsive Coulomb pseudopotential, μ*, significantly influence the superconducting transition temperature, *T*_c_. If the value of *N*(*E*_F_) decreases/increases with increasing pressure, then the value of *T*_c_ also decreases/increases with pressure [31,32,62]. Figure 8*a*,*b* shows the values of *N*(*E*_F_) and μ* for both compounds under applied pressure. From figure 8*a*,*b*, it is observed that the value of *N*(*E*_F_) and μ* of both compounds decreases slowly with rising pressure. This implies that the Coulomb electronic correlations become weaker with increasing pressure.

### 3.5. Chemical bonding

The Mulliken atomic population analysis provides significant insights into the bonding nature and electron charge density distribution in the SrPd₂P₂ and SrPd₂As₂ superconductors [63]. The calculated Mulliken atomic populations for the superconductors SrPd₂P₂ and SrPd₂As₂ at 0 and 16 GPa are provided in table 5. The data in table 5 indicate that in SrPd₂P₂, the P and Pd atoms carry a negative charge, while the Sr atoms have a positive charge, signifying charge transfer from Sr to P and Pd atoms. In the case of SrPd₂As₂, only Pd atoms bear a negative charge, with As and Sr atoms carrying a positive charge, indicating charge transfer from As and Sr atoms to Pd atoms. A low bond population value suggests an ionic character (where a bond population of zero represents a perfectly ionic bond), while values deviating from zero indicate increasing covalence [64]. Based on this, the P–P and P–Pd bonds in SrPd₂P₂ exhibit covalent characteristics, while Sr does not form any bonds. As the pressure increases to 16 GPa, the bond populations slightly increase and the bond lengths decrease, reflecting stronger covalent bonding under pressure without significant changes in the nature of the bonds.

**Table 5.** Mulliken atomic populations of SrPd₂P₂ and SrPd₂As₂ superconductors at 0 and 16 GPa.

compound	*P* (GPa)	species	*s*	*p*	*d*	total	charge	bond	population	length (Å)
SrPd_2_P_2_	0	P	1.60	3.44	0.00	5.05	−0.05	P–P	0.59	2.22239
		Pd	0.48	0.67	9.40	10.56	−0.56	P–Pd	0.78	2.50156
		Sr	1.91	5.97	0.92	8.79	1.21	—	—	—
	16	P	1.54	3.47	0.00	5.01	−0.01	P–P	0.69	2.12353
		Pd	0.48	0.72	9.40	10.59	−0.59	P–Pd	0.79	2.41912
		Sr	1.79	5.96	1.05	8.80	1.2	—	—	—
SrPd_2_As_2_	0	As	1.39	3.33	0.00	4.72	0.28	As–As	0.52	2.55242
		Pd	0.60	0.79	9.40	10.78	−0.78	As–Pd	0.17	2.55492
		Sr	2.18	5.97	0.84	8.99	1.01	—	—	—
	16	As	1.27	3.38	0.00	4.65	0.35	As–As	0.70	2.36283
		Pd	0.57	0.84	9.40	10.8	−0.81	As–Pd	0.02	2.46533
		Sr	2.13	5.95	1.00	9.08	0.92	—	—	—

Similarly, in SrPd₂As₂, the As–As and As–Pd bonds show covalent characteristics, and there is no bonding with Sr atoms. The bond populations do not change drastically at 16 GPa. However, the bond lengths decrease slightly with pressure, indicating stronger covalent bonds at higher pressure. The atomic population values do not change significantly at 16 GPa for both compounds, as shown in table 5. The bond lengths for P–P, P–Pd, As–As and As–Pd are also listed in table 5.

**Figure 9.**
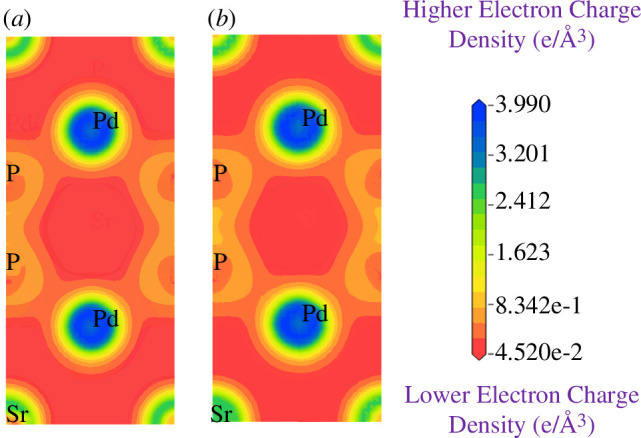
Electron charge density map of SrPd_2_P_2_ at (*a*) 0 GPa (*b*) 16 GPa.

**Figure 10.**
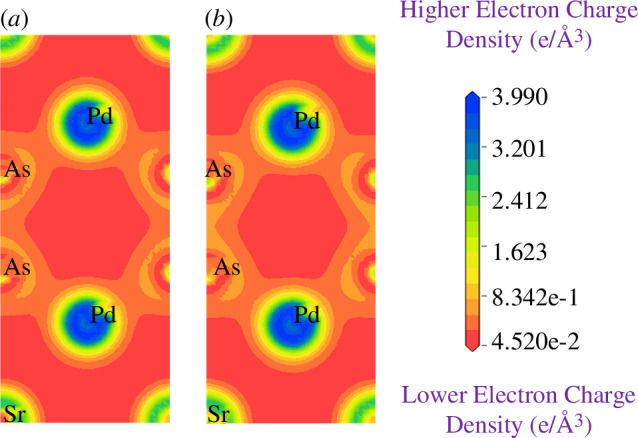
Electron charge density map of SrPd_2_As_2_ at (*a*) 0 GPa (*b*) 16 GPa.

To further support the explanation of the chemical bonding in SrPd₂P₂ and SrPd₂As₂ superconductors, the total electron density has been calculated using the conventional unit cell of both materials. Figures 9*a*,*b* and 10*a,b* illustrate the electron charge density distribution map for these superconductors at 0 and 16 GPa. A scale is shown on the right side of the contour plot, represented by a colour gradient in units of e Å^−^³. In this scale, blue colour indicates areas of high electron density, while red colour represents regions of low electron density. In figure 9*a*,*b* for SrPd₂P₂, the charge density overlap between P and Pd atoms suggests a covalent nature for the P–Pd bonds [65]. The shared charge between two P atoms clearly indicates the formation of a covalent P–P bond. Additionally, figure 9*a* shows no interaction between Sr and P or Sr and Pd atoms, confirming that Sr atoms do not form bonds in this structure. In figure 10*a*, for SrPd₂As₂, the covalent nature of the bonds is also evident due to the overlapping charge density between As and Pd atoms. The shared charge between two As atoms indicates the formation of a covalent As–As bond. No bonding is observed between Sr, As and Pd atoms, as there is no interaction between them. These strong covalent interactions in the P–P, P–Pd, As–As and As–Pd bonds define the electronic properties of these superconducting materials. At 16 GPa for both compounds (figures 9*b* and 10*b*) the charge density patterns do not change significantly, indicating that increased pressure does not substantially affect the bonding nature or electron sharing in this structure.

### 3.6. Optical properties

The response of a material to an incident electromagnetic wave can be understood through an analysis of its optical properties. In this regard, the optical properties of a material include different terms like reflectivity, absorption, dielectric function and conductivity [66,67]. In this study, the optical properties of SrPd_2_P_2_ and SrPd_2_As_2_ compounds were calculated for photon energies up to 20 eV, with a polarization vector along the direction under applied pressure. For a metallic compound, plasma energy is required for analysing the optical functions [24]. Plasma energy of 6 eV was used to study the optical functions of the SrPd_2_P_2_ and SrPd_2_As_2_ compounds.

**Figure 11.**
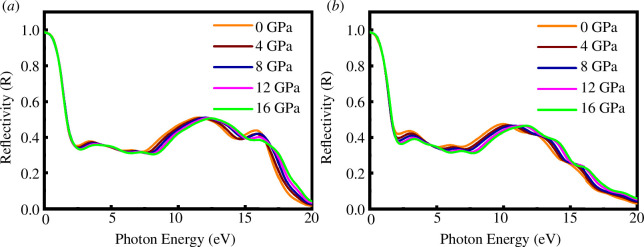
Optical function of reflectivity of (*a*) SrPd_2_P_2_ and (*b*) SrPd_2_As_2_ at various pressures.

The pressure-induced reflectivity spectra of SrPd_2_P_2_ and SrPd_2_As_2_ are represented in figure 11*a*,*b*. Figure 11*a,b* illustrates the reflectivity changes with photon energy. Reflectivity starts high (around 0.9) at low photon energies and decreases significantly as photon energy increases. There is a sharp drop in reflectivity around the 3–5 eV range. At higher photon energies (above 10 eV), the reflectivity curves converge, indicating reduced sensitivity to pressure changes. The fine structure and minor features around the 5–15 eV range vary slightly between the two graphs, indicating possible differences in the material’s response or experimental conditions. This result suggests that both compounds have the potential to be effective reflectors in a wide range of UV regions under applied pressure. It is also noticed that the reflectivity of both compounds does not significantly change with applied pressure.

**Figure 12.**
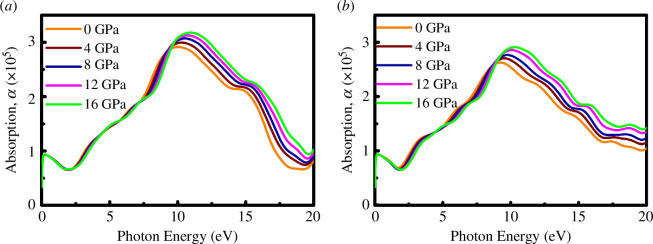
Optical function of absorption of (*a*) SrPd_2_P_2_ and (*b*) SrPd_2_As_2_ at various pressures.

The optical absorption coefficient can be used to measure the amount of light intensity that is attenuated as it passes through a material. Figure 12*a*,*b* displays the optical absorption coefficients of SrPd_2_P_2_ and SrPd_2_As_2_ compounds under applied pressure. From figure 12, it can be seen that the optical absorption of both compounds starts with zero photon energy, consistent with photoconductivity, dielectric function and band structure calculation. The SrPd_2_P_2_ and SrPd_2_As_2_ compounds show almost the same pattern under applied pressure up to 16 GPa in the visible region. After that, both compounds show an increase in absorption with photon energy, reaching a peak around 15 eV and then decreasing. In the UV region, the absorption coefficient has been determined to be more efficient, and the overall absorption coefficient of the SrPd_2_P_2_ compound is higher than that of SrPd_2_As_2_ under applied pressure. The separation between the absorption curves at higher energies suggests that the material’s absorption properties are more affected by pressure changes in this region.

**Figure 13.**
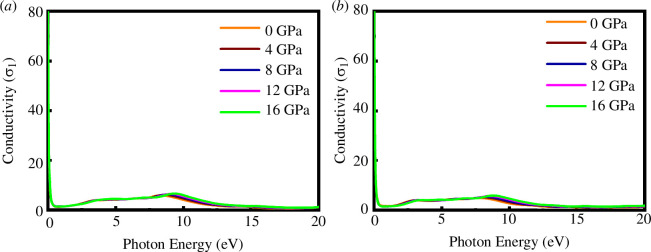
Real part of optical conductivity of (*a*) SrPd_2_P_2_ and (*b*) SrPd_2_As_2_ at various pressures.

**Figure 14.**
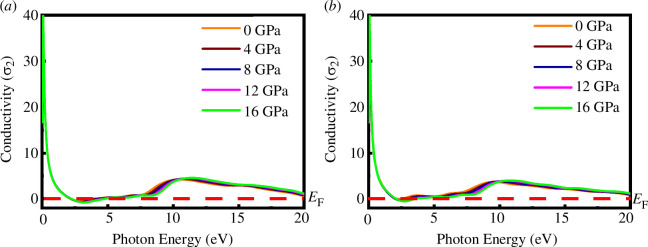
Imaginary part of optical conductivity of (*a*) SrPd_2_P_2_ and (*b*) SrPd_2_As_2_ at various pressures.

Figures 13*a,b* and 14*a,b* show the real and imaginary parts of optical conductivity for SrPd_2_P_2_ and SrPd_2_As_2_ compounds under various pressures as a function of photon energy, respectively. Both materials exhibit a high conductivity peak at low photon energies, quickly dropping off and remaining low with minor peaks around 2–3 and 10 eV. The real and imaginary conductivities do not change much when the pressure changes, indicating that the optical properties related to energy absorption of SrPd_2_P₂ and SrPd_2_As₂ are not significantly influenced by pressure within the studied range.

**Figure 15.**
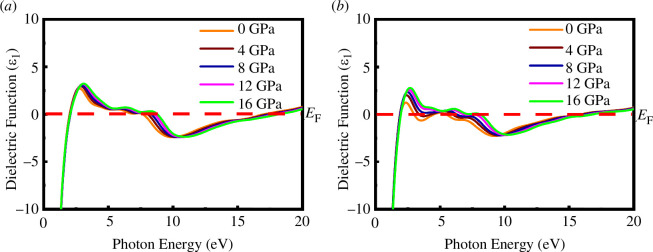
Real part of the dielectric function of (*a*) SrPd_2_P_2_ and (*b*) SrPd_2_As_2_ at various pressures.

**Figure 16.**
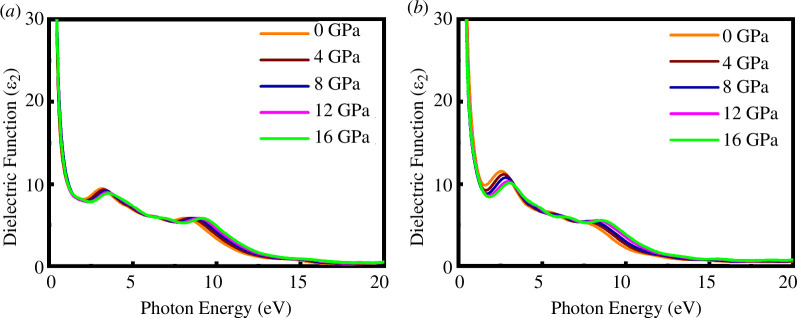
Imaginary part of the dielectric function of (*a*) SrPd_2_P_2_ and (*b*) SrPd_2_As_2_ at various pressures.

The real (*ε*_1_) and imaginary parts (*ε*_2_) of the dielectric function for both materials at different external pressures of up to 20 eV of photon energy are displayed in figures 15*a,b* and 16*a,b*. From figures 15 and 16, it can be observed that *ε*_1_ goes to zero from below at low photon energy, indicating the metallic nature, and the negative value of *ε*_1_ also reveals the Drude-like metallic nature of SrPd_2_P_2_ and SrPd_2_As_2_ compounds. The value of *ε*_2_ reaches approximately zero under higher photon energy, revealing that the SrPd_2_P_2_ and SrPd_2_As_2_ compounds behave as transparent in high-energy regions. The hydrostatic pressure does not significantly affect the dielectric functions of either compound. Pressure does not have a significant impact on the optical absorption curve, so dielectric functions are almost invariant under pressure.

### 3.7. Superconducting properties

The superconducting transition temperatures of SrPd_2_P_2_ and SrPd_2_As_2_ compounds at different pressures using the widely employed McMillan equation [68] are as given below,


(3.19) $$T_{c}=\;\frac{{ϴ}_{D}}{1.45}exp\left[-\frac{1.04\;\left(1+\lambda \right)}{\lambda -\mu^{*}\left(1+0.62\lambda \right)}\right],$$

where μ* and *λ* represent the Coulomb pseudopotential and electron–phonon coupling constants, respectively. The electron–phonon coupling constant can be represented in several forms, including: (i) through the electron–phonon spectral density, (ii) using the Hopfield parameter, the average phonon energy and the electronic density of states at the Fermi level, *N*(*E*_F_), and (iii) as the product of the electronic density of states at the Fermi level and the electron–phonon interaction energy. The third form has the simplest functional structure, where the electron–phonon coupling constant is given by *λ* = *N*(*E*_F_)*V*_e-ph_. Here, *V*_e-ph_ represents the electron–phonon interaction energy that leads to Fermi surface instability and Cooper pairing, which is a weakly varying function of pressure in most cases. *T*_c_ is determined by three physical parameters: the Debye temperature (*ϴ*_D_), the repulsive Coulomb pseudopotential (µ*) and the electron–phonon coupling constant (*λ*). Debye temperature increases with increasing pressure (table 4), while µ* decreases gradually with rising pressure (figure 8*b*) for both compounds. The value of *λ* can be expressed as *λ* = *N(E*_F_)*V*_e–ph_; any variation in the *V*_e–ph_ due to pressure has not been considered. Therefore, the changes in λ = *N(E*_F_)*V*_e–ph_ due to pressure become a linear function of *N*(*E*_F_). As the pressure increases, *N*(*E*_F_) experiences a decrease (figure 8*a*). This implies the decrement of *λ* with increasing pressure of SrPd_2_P_2_ and SrPd_2_As_2_ superconductors. Based on the pressure-dependent values of *ϴ*_D_, µ* and *λ,* it can be predicted that the value of *T*_c_ for SrPd_2_P_2_ and SrPd_2_As_2_ superconductors may decrease as pressure increases [31,32].

## 4. Conclusions

Using first-principles DFT-based calculations, the structural, mechanical, thermodynamic, electrical, optical and superconducting properties of the SrPd_2_P_2_ and SrPd_2_As_2_ superconductors with tetragonal crystal symmetry have been explored. The lattice constants and unit cell volume decrease almost linearly with increasing pressure, indicating a reduction in atomic spacing under hydrostatic pressure. Pugh’s ratio for both compounds indicates ductile behaviour under pressure. The universal anisotropy value demonstrates that SrPd₂P₂ and SrPd₂As₂ exhibit an anisotropic nature under applied pressure. SrPd_2_As_2_ has a lower *H*_v_ value compared with SrPd_2_P_2_ under pressure, indicating that SrPd_2_P_2_ is significantly harder. The values of *ϴ*_D_ are consistent with the average sound velocity, with both these parameters increasing as pressure rises. The *T*_m_ of both compounds also increases with pressure, and at 16 GPa, SrPd₂P₂ exhibits a higher *T*_m_ than SrPd₂As₂, indicating stronger bonding and greater thermal stability in SrPd₂P. The studies of the electronic band structure and DOS values confirm the metallic nature of SrPd_2_P_2_ and SrPd_2_As_2_ compounds. In the visible spectrum, both superconductors exhibit a prominent reflectivity peak, while they function as efficient absorbers in the UV region. The optical conductivity of both compounds starts at zero photon energy, which is an expected behaviour for a metallic system. As pressure increases, the electronic band structure near the Fermi level becomes more dispersive, resulting in a decrease in *N*(*E*_F_) and µ*. Consequently, *λ* also decreases with rising pressure, as it becomes a linear function of *N*(*E*_F_). This suggests that the *T*_c_ of both superconductors may decrease under applied pressure.

## Data Availability

The data is available from the corresponding authors upon reasonable request.
